# DNA methylation levels at individual age-associated CpG sites can be indicative for life expectancy

**DOI:** 10.18632/aging.100908

**Published:** 2016-02-25

**Authors:** Qiong Lin, Carola I. Weidner, Ivan G. Costa, Riccardo E. Marioni, Marcelo R. P. Ferreira, Ian J. Deary, Wolfgang Wagner

**Affiliations:** ^1^ Helmholtz-Institute for Biomedical Engineering, Stem Cell Biology and Cellular Engineering, RWTH University Medical School, Aachen, Germany; ^2^ Institute for Biomedical Technology – Cell Biology, RWTH University Medical School, Aachen, Germany; ^3^ Interdisciplinary Centre for Clinical Research (IZKF), RWTH University Medical School, Aachen, Germany; ^4^ Centre for Cognitive Ageing and Cognitive Epidemiology, University of Edinburgh, Edinburgh EH8 9JZ, UK; ^5^ Medical Genetics Section, Centre for Genomic and Experimental Medicine, Institute of Genetics and Molecular Medicine, University of Edinburgh, Edinburgh EH4 2XU, UK; ^6^ Queensland Brain Institute, The University of Queensland, Brisbane 4072, QLD, Australia; ^7^ Department of Psychology, University of Edinburgh, Edinburgh EH8 9JZ, UK; ^8^ Department of Statistics, Centre for Natural and Exact Sciences, Federal University of Paraiba, CEP 58051-900, João Pessoa, Brazil

**Keywords:** DNA-methylation, epigenetic, aging, age, prediction, predictor, survival, mortality, PDE4C, CLCN6

## Abstract

DNA-methylation (DNAm) levels at age-associated CpG sites can be combined into epigenetic aging signatures to estimate donor age. It has been demonstrated that the difference between such epigenetic age-predictions and chronological age is indicative for of all-cause mortality in later life. In this study, we tested alternative epigenetic signatures and followed the hypothesis that even individual age-associated CpG sites might be indicative for life-expectancy. Using a 99-CpG aging model, a five-year higher age-prediction was associated with 11% greater mortality risk in DNAm profiles of the Lothian Birth Cohort 1921 study. However, models based on three CpGs, or even individual CpGs, generally revealed very high offsets in age-predictions if applied to independent microarray datasets. On the other hand, we demonstrate that DNAm levels at several individual age-associated CpGs seem to be associated with life expectancy – e.g., at CpGs associated with the genes *PDE4C* and *CLCN6*. Our results support the notion that small aging signatures should rather be analysed by more quantitative methods, such as site-specific pyrosequencing, as the precision of age-predictions is rather low on independent microarray datasets. Nevertheless, the results hold the perspective that simple epigenetic biomarkers, based on few or individual age-associated CpGs, could assist the estimation of biological age.

## INTRODUCTION

Aging is associated with highly reproducible DNA-methylation (DNAm) changes at specific sites in the genome [1-5]. Various combinations of age-associated CpG dinucleotides have been used for age-estimation and the average absolute difference of DNAm-predicted and chronological age (Δ_age_) can be less than five years [6-8]. While such epigenetic aging signatures are usually trained to be as precise as possible, the Δ_age_ can partly be attributed to effects of biological aging. Recently, it has been demonstrated that Δ_age_ is indicative for life expectancy: in four longitudinal cohorts of older people, accelerated epigenetic aging was associated with higher all-cause mortality [9]. That study utilized two independent aging signatures: a predictor by Hannum and coworkers [6] based on 71 CpGs, and an “epigenetic clock” by Horvath that utilizes 353 CpGs [7]. However, using our previously described model based on three CpG sites (associated with genes *ASPA*, *ITGA2B* and *PDE4C*) [8] there was no clear correlation with chronological age and therefore this 3-CpG model was not further considered [9].

Simple aging signatures - based on few or even individual CpGs - facilitate site-specific analysis with more quantitative methods without need of profiling technology. When we apply the 3-CpG model on pyrosequencing data of blood samples the median Δ_age_ is usually about 5 years [8,10]. The discrepancy to the above mentioned study [9] can partly be attributed to the fact that the 3-CpG model was not trained on Illumina HumanMethylation450 BeadChip data and that it involves a neighbouring CpG site not measured by these microarrays. Therefore, we have now adjusted the 3-CpG model to Illumina HumanMethylation450 BeadChip data to test it again on the dataset of the Lothian Birth Cohort 1921 study (LBC1921). If such concise age-predictors are associated with life expectancy, they might provide convenient and cost-effective biomarkers for biological age.

## RESULTS AND DISCUSSION

### Epigenetic aging-signatures are more robust if considering more CpGs

**Figure 1:**
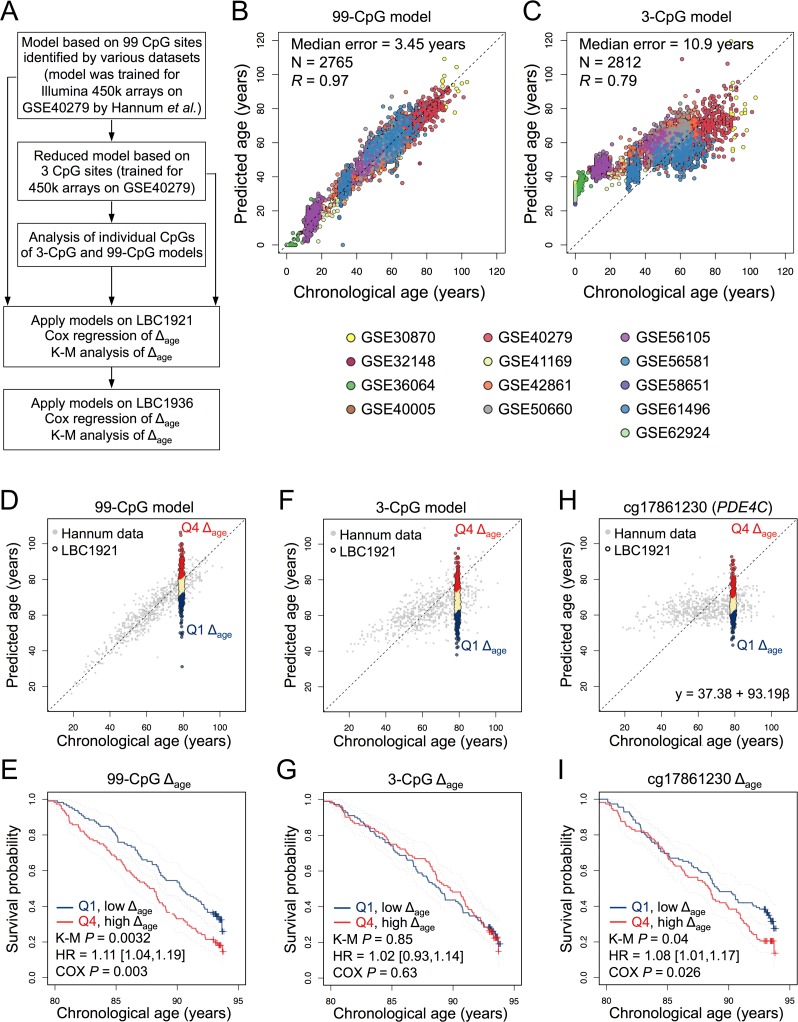
Epigenetic age-predictions correlate with mortality **(A)** Scheme for the study design. Epigenetic age was estimated based on our previously published models with 99 CpGs, 3 CpGs, or individual CpGs thereof [8]. The offset of predicted age and chronological age was subsequently correlated with all-cause mortality in the Lothian Birth Cohort 1921 (LBC1921) and LBC1936. **(B)** The 99-CpG model and **(C)** the 3-CpG model were specifically trained for Illumina HumanMethylation450 BeadChip data using Hannum dataset (GSE40279) and subsequently validated using additional 12 DNAm datasets of blood samples. **(D)** The 99-CpG model was then applied on DNAm profiles of LBC1921 and the deviation of predicted and chronological age (Δ_age_) was determined for each sample. Samples in the lowest (Q1) and highest quartiles (Q4) of Δ_age_ are depicted in navy and red, respectively. **(E)** Kaplan-Meier plots (K-M) of LBC1921 participants classified by respective quartiles indicate lower mortality for those with increased Δ_age._. **(F, G)** In analogy the same analysis was performed for the 3-CpG model, but there was no significant association between these age-predictions and mortality. **(H, I)** Alternatively, we tested association of DNAm at individual age-associated CpGs with mortality. To this end, linear models were trained for each CpG site using Hannum dataset and then applied to the LBC1921 cohort. cg17861230 (associated with *PDE4C*) reveals a significant association with mortality. The calculated Δ_age_ is subject to survival analysis with adjustment for chorological age and gender.

For an independent aging-signature based on multiple CpGs we used our previously described model based on 99 age-associated CpGs (99-CpG model; Figure 1A) [8,11]. This model was initially derived from HumanMethylation27 BeadChip data [8] and subsequently trained on 656 DNAm profiles of blood samples [6]. The coefficients for this model are provided in Supplementary Table 1. To estimate the validity of this model, we have tested over ∼2,100 DNAm profiles from 12 additional studies (Supplementary Table 2): overall, there was a high correlation of predicted and chronological age (Pearson correlation *R* = 0.97; median error = 3.45 years; Figure 1B). Thus, this relatively large signature of 99 CpGs can be applied to DNAm profiles without need of an additional normalization regimen.

Similarly, we compared the performance of our 3-CpG model [8] - as an example for a simple age-predictor - on independent microarray datasets. This model was initially trained on pyrosequencing data and it was therefore retrained on the above mentioned dataset of 656 DNAm profiles [6]. Thereby, we derived the following multivariate 3-CpG model for Illumina HumanMethylation450 BeadChip profiles:

Predicted age (in years) = 111.83 - 64.57[β-value cg02228185] - 42.57 [β-value cg25809905] + 75.15 [β-value cg17861230].

In the validation set of ∼2,100 DNAm profiles the correlation with chronological age was R = 0.79 and median error was 10.9 years (Figure 1C). Thus, the precision of this adjusted 3-CpG model is better than before [9], but still not in the range of predictions for pyrosequencing data (median Δ_age_ ≈ 5 years). Notably, samples of younger donors were more likely to be overestimated in their epigenetic age. This might be partly attributed to the fact that the training datasets did not comprise samples of children. Furthermore, it has been demonstrated that many DNAm changes are not linearly acquired over childhood [12]. DNAm patterns vary between cell types and therefore blood counts may affect age-predictions – albeit we have previously demonstrated that the composition of different blood cell types has relatively little impact on predictions by our 3-CpG model [8]. Accuracy can be improved by using additional CpGs (Supplementary Figure 1). Another possibility is to normalize the DNAm profiles – but this would again necessitate DNAm levels of a multitude of additional CpGs.

Advantages of age-predictors based on few or individual CpGs are that (i) they can be measured site-specifically with quantitative and cost-effective methods, (ii) they can be applied with less bioinformatic knowledge, and (iii) they are independent from specific microarray platforms. The importance of the latter becomes evident by the fact that Illumina has recently announced to replace the HumanMethylation450 BeadChip with a new platform. On the other hand, our analysis exemplarily demonstrates that age-predictors based on few CpGs are less precise in cross-comparison of different studies if applied to β-values of microarray data. Although simple age-predictors reveal higher deviation of chronological age in microarray data, their Δ_age_ might still be indicative for overall survival.

### Δ_age_ is indicative for life expectancy

The 99-CpG model and the adjusted 3-CpG model were subsequently applied to DNAm profiles of the LBC1921 study [9,13]. These participants were born in 1921 and recruited and tested in older age between 1999 and 2001 (N = 446; n_death_ = 328). The 99-CpG model and 3-CpG model revealed median error of 5.3 and 11.5 years, respectively (Table 1) – whereas it was 5.5 and 6.0 years using the age-predictors by Hannum et al. or Horvath [9]. In the 99-CpG model, a five-year higher age-prediction was associated with 11% greater mortality risk (95% confidence interval: [1.04, 1.19]; Cox regression *P* = 0.003) after adjustment for gender and chronological age. Kaplan-Meier (K-M) analysis of quartiles with highest and lowest Δ_age_ (*P* = 0.0032) further visualized and validated that epigenetic-age predictions are indicative for all-cause mortality (Figure 1D,E).

**Table 1:** Age-prediction of the two mortality cohorts

Lothian Birth Cohorts	LBC1921	LBC1936
N	446	920
n (death)	328	135
Age (years ± S.D.)	79.1 ± 0.6	69.5 ± 0.8
Sex (male)	176 (40%)	465 (51%)
99CpG DNAm Age (years ± S.D.)	76.6 ± 8.8	66.4 ± 9.4
99CpG Δ_age_ (years ± S.D.)	−2.5 ± 8.8	−3.2 ± 9.1
99CpG median error (years)	5.3	5.4
99CpG Δ_age_ HR [95% CI]	1.11 [1.04,1.19]	1.02 [0.93,1.14]
3CpG DNAm Age (years ± S.D.)	68.4 ± 9.9	61.2 ± 8.9
3CpG Δ_age_ (years ± S.D.)	−10.7 ± 10	−8.3 ± 9.0
3CpG median error (years)	11.5	8.6
3CpG Δ_age_ HR [95% CI]	1.01 [0.96,1.08]	1.02 [0.92,1.13]

When we used the 3-CpG signature this association was not significant (Hazard ratio [95% CI] of Cox regression: 1.02 [0.93,1.14]); Figure 1F,G). This might be due to the higher offset in age-predictions. Overall, the LBC1921 samples were underestimated in their epigenetic age and the median error was relatively high. It is also conceivable that some age-associated CpGs reflect life expectancy better than others. Therefore, we trained linear models for each of the three age-associated CpGs individually and tested association of their Δ_age_ with life-expectancy. For the CpG associated with the phosphodiesterase 4C (*PDE4C;* cg17861230) we found a significant association with overall survival (HR = 1.08 [1.01; 1.17]; Cox *P* = 0.026; Figure 1H,I) but not for the other two CpGs. Subsequently, we tested all individual CpGs of the three larger signatures: our 99-CpG model, the age-predictor by Hannum et al. [6], and of Horvath [7]. Although, the underlying algorithms are based on the combinatorial effect of multiple age-associated CpGs, we identified 5 (of 99), 10 (of 71), and 0 (of 353) significant CpGs, which are highly associated with mortality, respectively (Cox *P* < 0.05, multiple correction testing by the Benjamini-Hochberg procedure; Supplementary Tables 3-5).

### Comparisons in LBC1921 and LBC1936

To further validate mortality-association of these CpGs we used DNAm profiles of the first wave in the Lothian Birth Cohort 1936 study (LBC1936). These participants were analyzed at an average age of 70 years (N = 920; n_death_ = 135). However, neither the 99-CpG model nor the 3-CpG model revealed significant association with mortality, which might be due to the relatively low number of deaths in this cohort (Table 1). On the other hand, age-predictions by the models of Hannum and coworkers and of Horvath have previously been demonstrated to be indicative for all-cause mortality in the LBC1936 dataset [9].

Subsequently, we tested for associations of single-CpG derived age-predictions with mortality: several CpGs revealed significant results in the LBC1936 data but there was only a moderate overlap with survival-associated CpGs in LBC1921 (Supplementary Tables 3-5). For example, the CpG site located in *PDE4C* (cg17861230) revealed a similar trend but the results were not significant (HR = 1.09 [0.98; 1.21]; Cox *P* = 0.12; Supplementary Figure 2). It might be expected that DNAm changes at these CpGs are not entirely linear over time and hence they may have different prognostic value in cohorts of different age.

**Figure 2:**
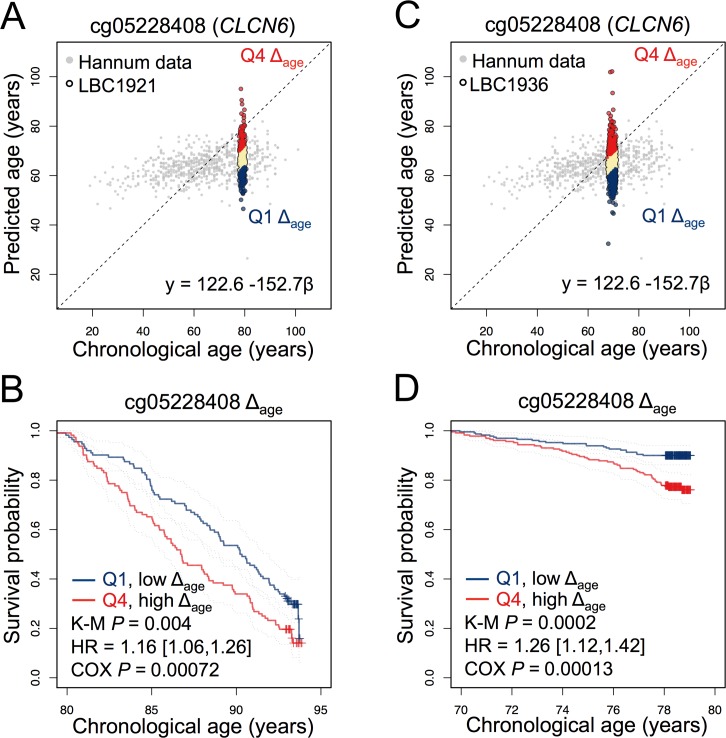
A CpG site in CLCN6 is indicative for survival in LBC1921 and LBC1936 **(A)** A CpG site associated with the gene for the chloride transport protein 6 (*CLCN6;* cg05228408) was used to estimate age in the LBC1921 cohort. **(B)** Participants in the lowest and highest quartiles of Δ_age_ were subsequently analysed in Kaplan-Meier plots (K-M). Hazard ratio and cox-regression analysis of survival were subsequently tested with adjustment for chorological age and gender. **(C, D)** In analogy, the same analysis was performed for this CpG site using the LBC1936 cohort.

Only one of the tested CpG sites (from the 99-CpG model) revealed significant association with survival in the LBC1921 and LBC1936 datasets. It was associated with the gene for the chloride transport protein 6 (*CLCN6;* cg05228408) and we observed significant association in LBC1921 (HR = 1.16 [1.06,1.26]; Cox *P* = 0.00072) and LBC1936 (HR = 1.26 [1.12,1.42]; Cox *P =* 0.00013) after multiple correction and adjustment for age and gender in each cohort (Figure 2). Notably, several studies identified single nucleotide polymorphisms in vicinity to this location that are associated with blood pressure and hypertension [14-16]. Furthermore, a genome-wide association study in rats supported the notion that multiple modifiers of hypertension co-segregate at this locus [17]. We have checked in both LBC cohorts if DNAm at *CLCN6* is directly associated with hypertension, but the correlation with systolic and diastolic blood pressure was low (Spearman correlation: R = −0.022 and 0.013, respectively). Other parameters, such as specific drugs, smoking, and alcohol intake might also affect DNAm. Either way, β-values at this age-associated CpG seem to be associated with life expectancy – this should be validated by site-specific analysis (e.g. by pyrosequencing of bisulfite converted DNA) in a suitable cohort in the future.

## CONCLUSIONS

This follow-up study further substantiates the notion that epigenetic age-predictions are indicative for biological rather than chronological age [9,18]. Similar findings have recently been described in a longitudinal Danish twin study [19]. We demonstrated that the error of age-predictions can be improved for the 3-CpG model by training on HumanMethylation450 BeadChip data. However, without additional normalization regimen, such small aging signatures are not reliable for microarray data – they should rather be addressed by more quantitative methods for site-specific analysis such as pyrosequencing or MassARRAY. On the other hand, we demonstrate that even β-values at individual age-associated CpGs seem to be indicative for life expectancy.

Microarray data of genome wide DNAm profiles of large cohort studies resemble a valuable resource to correlate DNAm patterns with clinical parameters – however, if there is a systematic off-set in epigenetic age-predictions, resulting in a different slope in comparisons of predicted and chronological ages, then this will falsify association with life-expectancy, because the percentage of elderly patients that are predicted to be older than their chronological age is affected. Such a confounding factor is particularly relevant for the small aging-signatures that are generally more likely to reveal offsets in other microarray datasets – but it was hardly relevant in our exemplary analysis, as we only considered the first waves of the LBC1921 and LBC1936 datasets with well-defined donor ages close to 79 and 70 years, respectively. To ultimately validate the association of simple epigenetic biomarkers with biological age, it will be necessary to utilize site-specific methods for DNAm analysis – so far DNAm results by pyrosequencing are not available for large cohorts with adequate information on life-expectancy and other clinical parameters. Our study provides the research perspective that site-specific analysis of individual age-associated CpG sites can facilitate cost-effective high throughput analysis to better discern environmental or genetic risk factors to improve the odds of staying healthy.

## METHODS

### The Lothian Birth Cohorts

The Lothian Birth Cohorts of 1921 and 1936 are follow-up studies of the Scottish Mental Surveys of 1932 and 1947 – for participants born in 1921 and 1936, respectively. These nationwide studies were initially set up to study determinants of non-pathological cognitive ageing [13]. The LBC1921 and LBC1936 studies attempted to follow-up individuals in the Lothian region (Edinburgh and its surrounding areas of Scotland) at about the age of 79 years and 70 years, respectively. There have been various additional follow up waves at higher ages but we restricted our analysis to the first waves to facilitate better comparison with the previous analysis [9] and to exclude effects that might be caused by offsets in the regression of age-predictions or by repeated analysis of the same individuals.

### Ethics and data deposition

Ethics permission for LBC1921 was obtained from the Lothian Research Ethics Committee (Wave 1: LREC/1998/4/183) and for LBC1936 from the Multi-Centre Research Ethics Committee for Scotland (Wave 1: MREC/01/0/56). Written informed consent was obtained from all subjects. The data have been deposited at the European Genomephenome Archive (EGA; http://www.ebi.ac.uk/ega/ home) under the accession number EGAS00001000910.

### DNA methylation of LBC cohorts

The DNAm data were processed as previously described [9]. Briefly, raw DNAm data of LBC cohort (LBC1921 N = 514; LBC1936 N = 1,004) were background corrected and converted to methylation β-values using the R minfi package (β-values range between 0 and 1 and roughly correspond to 0% and 100% DNAm level, respectively). The probes with a low (<95%) detection rate at P <0.01 were removed from further analysis. In addition, manual inspection of the array control probe signals was used to identify and remove low quality samples, resulting two high quality datasets for aging prediction (LBC1921: N = 443; LBC1936: N = 920; all samples are from first wave).

### Derivation of age predictors

Our 99-CpG model and our 3-CpG model for epigenetic age-predictions was initially derived from 102 CpGs that revealed linear age-associated changes in 575 DNAm profiles of blood that were generated on Illumina HumanMethylation27 BeadChips (Pearson correlation R > 0.85 or R < −0.85; age range 0 to 78 years) [8]. Ninety-nine of these CpGs are also represented on the Illumina Human-Methylation450 BeadChips. Derivation of the 99-CpG model has been described in detail before [8,11] and the coefficients are provided in Supplementary Table 1. The 3-CpG model was based on β-values of the CpG sites cg02228185 (ASPA), cg25809905 (ITGA2B), and cg17861230 (PDE4C). It was retrained on the 656 DNAm profiles of blood samples of Hannum and coworkers (age range 19 to 101) [6] using leave-one-out cross validation. Age predictors based on individual CpGs were also trained on the dataset by Hannum et al. using leave-one-out cross validation. Please note that using β-values of the corresponding CpGs would have provided similar results as the participants of the waves 1 were all of similar age. The analysis were performed using R [20] and ‘caret’ package [21].

### Training and validation of the epigenetic age-predictors

To validate the 99-CpG and 3-CpG model (trained using dataset by Hannum et al. [6]; GSE40279) we used 12 additional publically available DNAm datasets of blood that were retrieved from NCBI GEO: GSE30870 [22], GSE32148 [23], GSE36064 [12], GSE40005, GSE41169 [24], GSE42861 [25], GSE50660 [26], GSE56105 [27], GSE56581 [28], GSE58651 [29], GSE61496 [30] and GSE62924 [31] (Supplementary Table 2). These validation datasets were all generated using Illumina HumanMethylation450 BeadChip.

### Survival analysis

The association of Δ_age_ and mortality was tested using cox proportional hazard regression models, adjusting for age and gender. The deaths within the first 2 years of follow-up were excluded to minimize the potential influences of acute illness when cox regression analysis is applied for the Δ_age_ of 99-CpG and 3-CpG models [9]. Hazard ratios for Δ_age_ were expressed per 5 years of methylation age acceleration as previously described [9]. For Kaplan-Meier (K-M) estimation of mortality samples were stratified by first and forth quantile of Δ_age_ adjusting for age and gender. The analysis were performed using R and ‘survival’ package [32].

## SUPPLEMENTARY DATA


