# Supplementary material for: DNA methylation levels at individual age-associated CpG sites can be indicative for life expectancy
Source: Aging (Albany NY). 2016 Feb 25;8(2):394–401. doi: 10.18632/aging.100908 (PMC4789590; doi:10.18632/aging.100908)
Supplement: Supplementary file 1 [file aging-08-394-s001.pdf]

SUPPLEMENTARY DATA

DNA methylation levels at individual age-associated CpG sites can be indicative for life expectancy.

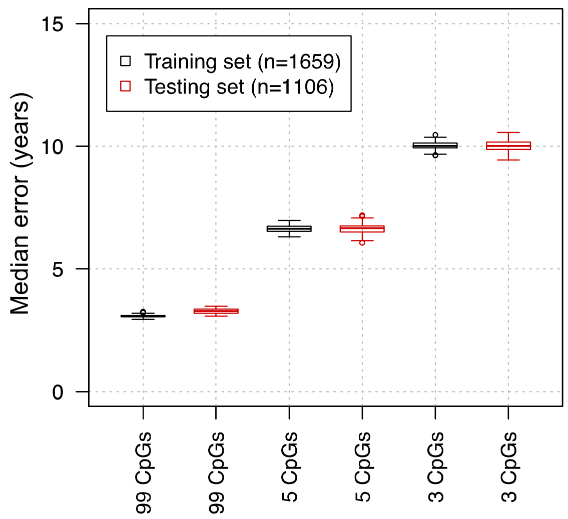

Supplementary Figure 1. Validation of the 99-CpG and 3-CpG models using multiple published datasets.
Age-prediction were calculated in 2,765 DNAm profiles of blood samples (the 13 datasets are provided in Supplemental Table 2). DNAm profiles were iteratively separated into training sets (60% of all samples; for building linear models using leave-one-out cross validation) and testing sets (40%) for validation (performed 100 times). Models were calculated for the three CpGs of the 3-CpG model; for five CpGs that included two additional age-associated CpGs from our previous study (cg09809672, *EDARADD*; and cg15279633, *RAB36*), or all 99 CpGs of the 99-CpG model.

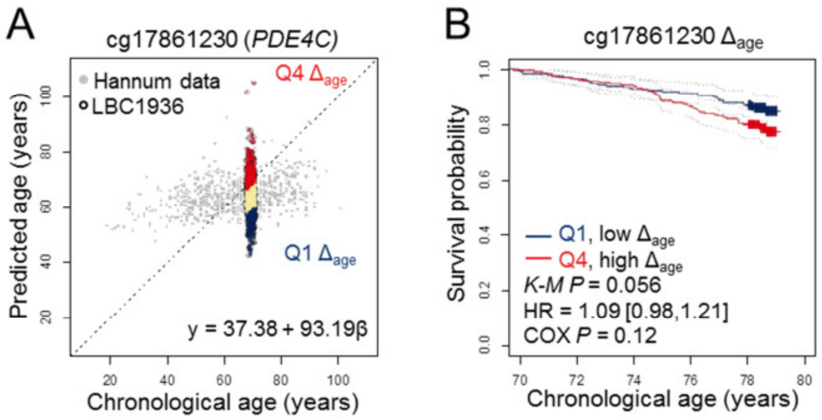

Supplementary Figure 2. CpG site cg17861230 (*PDE4C*) with mortality correlation in LBC1936.
(A) The linear model for the CpG site in *PDE4C* was applied to the LBC1936 cohort and **(B)** the resulting Δ_{age} was correlated with survival data (in analogy to Figure 1H, I). DNAm at this CpG site revealed a similar trend as observed in the LBC1921 study, but the results were not significant. This might be due to the relatively low number of deaths in this cohort.

Supplementary Table 1: Coefficients for the 99-CpG model.

CpG sites	Coefficient
(Intercept)	12.2169841
cg05228408	0.47636173
cg16352283	-5.3124138
cg05436231	17.7305146
cg19046959	-13.367066
cg17791651	8.72680959
cg07388493	-4.7759575
cg04036898	10.162153
cg07810156	15.3892025
cg21448423	-4.4621797
cg18660898	13.2543665
cg25256723	-11.802998
cg21870884	22.9981412
cg25947945	-8.2387336
cg09462576	6.3124836
cg09809672	-14.950409
cg27553955	-0.7884001
cg27320127	48.9368049
cg15297650	-34.306553
cg05331214	9.83640629
cg24178740	-27.476107
cg18182399	-4.1527608
cg25431974	-1.048605
cg24768561	-4.5917403
cg26614073	-11.443446
cg23320649	8.70555476
cg12554573	1.81880164
cg04474832	-26.556597
cg17421623	2.4399993
cg22919728	0.99214006
cg14456683	13.1457167
cg08209133	30.500322
cg16744741	-9.5846721
cg00059225	36.8195086
cg00489401	1.98682848
cg02844545	0.38022482
cg22736354	36.9317174
cg06493994	66.1611861
cg03340878	5.95485236
cg03958979	-16.016804
cg15804973	-15.214138
cg13870866	-39.104364
cg00503840	31.2113275
cg25762706	1.5340163
cg25538571	10.2956593
cg08598221	2.62080161
cg19724470	-5.5537073
cg07211259	-12.424324
cg13870494	19.7417678
cg16386080	-29.24993
cg00563932	-3.5009711
cg21120249	-8.6074197
cg26581729	-7.9914389
cg17431739	8.22589722
cg13129046	-5.1368284
cg01560871	13.5034883
cg06291867	13.0769424
cg26610808	-21.374356
cg07621046	13.6468199
cg13807496	-8.3931276
cg20654468	8.14605552
cg21992250	10.5216611
cg15538427	-19.93487
cg08012287	-18.989957
cg01820374	-30.896866
cg19722847	-13.06341
cg12883767	8.45912249
cg04123409	-10.767354
cg22580512	-0.8383178
cg25268718	4.06576438
cg21296230	28.1787443
cg21801378	44.7163476
cg10917602	-6.0293979
cg15195412	20.050343
cg20264732	-20.618882
cg22947000	-13.217155
cg02228185	-8.6363427
cg01739167	33.8101434
cg14918082	15.5554908
cg05379350	17.340667
cg08468689	-16.062905
cg08090640	8.31318309
cg25809905	-6.0974732
cg05294455	2.71073045
cg06638433	10.6229217
cg20366832	2.97899616
cg19761273	-16.331359
cg26927807	16.5195276
cg17471102	-18.063487
cg02489552	6.09699424
cg05488632	-11.249025
cg16363586	13.6452671
cg17861230	17.5027126
cg24713204	-32.487323
cg23679724	0.25793126
cg03224418	8.07556639
cg15379633	15.4139903
cg02994956	-6.4516149
cg23124451	-13.361462
cg26394940	0.89292205

Supplementary Table 2: Additional information on publically available GEO datasets used in this study.

GEO ID	Tissue (cell types)	Number of DNAm profiles	Age range	Mean age
GSE40279	Whole blood	656	19-101	64
GSE30870	Whole blood/cord blood	40	0-103	47
GSE32148	Peripheral blood	20	3-76	23
GSE36064	Leukocytes	78	1-16	5
GSE40005	Blood	12	53-68	61
GSE41169	Whole blood	33	18-65	29
GSE42861	Peripheral blood leukocytes	335	20-70	53
GSE50660	Peripheral blood	464	38-67	55
GSE56105	Peripheral blood lymphocytes	614	10-75	21
GSE56581	CD4+ cells	214	45-79	59
GSE58651	Peripheral blood	3	0	0
GSE61496	Whole blood	312	30-74	49
GSE62924	Cord blood leukocytes	38	0	0

All datasets have been generated on 450k Illumina BeadChips. Only the data from heathy donors were included in the analysis. Due to missing values some profiles had to be excluded.

Supplementary Table 3: CpGs of the 99-CpG model that correlate with life expectancy.

Name	Gene Symbol	LBC1921 COX *P*-value	LBC1921 COX adjusted *P*-value	LBC1936 COX *P*-value	LBC1936 COX adjusted *P*-value
cg12554573	*PARP3*	**1.1400E-05**	**0.0011**	0.0667	0.2923
cg25268718	*PSME1*	**0.0001**	**0.0070**	**0.0248**	0.1870
cg05228408	*CLCN6*	**0.0007**	**0.0235**	**0.0001**	**0.0129**
cg03224418	*SAMD10*	**0.0010**	**0.0241**	0.1770	0.4927
cg04474832	*ABHD14B*	**0.0024**	**0.0461**	0.4220	0.6893
cg00563932	*PTGDS*	**0.0060**	0.0973	0.8300	0.9492
cg26581729	*NPDC1*	**0.0074**	0.1033	**0.0173**	0.1870
cg05379350	*GIT1*	**0.0085**	0.1033	0.1750	0.4927
cg25947945	*LAD1*	**0.0096**	0.1033	0.3420	0.6464
cg15538427	*LRRN4CL*	**0.0107**	0.1033	0.0824	0.3365
cg21801378	*BRUNOL6*	**0.0116**	0.1033	0.9310	0.9748
cg20654468	*LPXN*	**0.0134**	0.1040	**0.0113**	0.1531
cg08468689	*GHDC*	**0.0138**	0.1040	0.0544	0.2666
cg23320649	*C3orf18*	**0.0171**	0.1197	**0.0330**	0.2156
cg23679724	*CTSZ*	**0.0194**	0.1267	0.4150	0.6893
cg17421623	*KTELC1*	**0.0243**	0.1437	**0.0098**	0.1531
cg26610808	*BLOC1S2*	**0.0256**	0.1437	0.4190	0.6893
cg17861230	*PDE4C*	**0.0264**	0.1437	0.1230	0.4305
cg21120249	*C9orf139*	**0.0290**	0.1490	**0.0237**	0.1870
cg08090640	*IFI35*	**0.0304**	0.1490	0.3830	0.6773
cg19761273	*CSNK1D*	**0.0323**	0.1507	0.2560	0.5575
cg26614073	*SCAP*	**0.0342**	0.1523	0.3270	0.6464
cg15297650	*TMEM163*	**0.0362**	0.1542	0.3850	0.6773
cg15195412	*CX3CL1*	**0.0390**	0.1560	**0.0229**	0.1870
cg20264732	*ESRP2*	**0.0398**	0.1560	**0.0291**	0.2037
cg16363586	*BST2*	**0.0471**	0.1720	**0.0022**	0.0836
cg12883767	*SLC26A10*	**0.0474**	0.1720	0.4650	0.7350
cg10917602	*HSD3B7*	**0.0497**	0.1740	0.6080	0.8162
cg15804973	*MAP3K5*	0.1120	0.2888	**0.0125**	0.1531
cg17791651	*POU3F1*	0.1300	0.3080	**0.0498**	0.2569
cg07388493	*NDUFS5*	0.1530	0.3487	**0.0026**	0.0836
cg25256723	*F5*	0.2660	0.5214	**0.0357**	0.2187
cg13870494	*MAMDC2*	0.7310	0.8736	**0.0095**	0.1531
cg19724470	*CD274*	0.7860	0.8921	**0.0224**	0.1870
cg21448423	*ACOT11*	0.7860	0.8921	**0.0464**	0.2526
cg07621046	*C10orf82*	0.8340	0.9081	**0.0414**	0.2387
cg02489552	*CCDC105*	0.9940	0.9940	**0.0084**	0.1531

Age-predictors were calculated for each of the 99 CpGs of our 99-CpG model and Δ_{age} was used for cox linear regression analysis of overall survival in either the LBC1921 or the LBC1936 study. *P*-values are provided with or without adjustment for multiple testing (99 CpGs) and significant CpGs are highlighted in bold ($P < 0.05$).

Supplementary Table 4: CpGs of the age-predictor by Hannum et al. that correlate with life expectancy.

**Name**	**Gene Symbol**	**LBC1921 COX *P*-value**	**LBC1921 COX adjusted *P*-value**	**LBC1936 COX *P*-value**	**LBC1936 COX adjusted *P*-value**
cg02085953	*ARID5A*	**4.0700E-05**	**0.0028**	0.4460	0.7615
cg08234504		**0.0002**	**0.0057**	0.3800	0.7613
cg04400972	*TRIM45*	**0.0002**	**0.0057**	0.7610	0.9529
cg03032497		**0.0004**	**0.0064**	0.7050	0.9457
cg00486113	*PSORS1C1*	**0.0005**	**0.0065**	0.9870	0.9870
cg07553761	*TRIM59*	**0.0006**	**0.0065**	0.5750	0.8385
cg22454769	*FHL2*	**0.0010**	**0.0100**	0.2730	0.7613
cg24079702	*FHL2*	**0.0016**	**0.0136**	0.5260	0.8182
cg04474832	*ABHD14B*	**0.0024**	**0.0183**	0.4220	0.7613
cg21139312	*MSI2*	**0.0064**	**0.0448**	**0.0241**	0.3500
cg23500537		**0.0082**	0.0523	0.2370	0.7517
cg07082267		**0.0135**	0.0788	0.7910	0.9529
cg08415592	*APOL1*	**0.0205**	0.1104	0.4000	0.7613
cg03399905	*ANKRD34C*	**0.0251**	0.1255	0.8710	0.9678
cg14692377	*SLC6A4*	**0.0274**	0.1279	0.1090	0.5119
cg19935065	*DNTT*	**0.0360**	0.1575	0.2940	0.7613
cg08097417	*KLF14*	**0.0414**	0.1705	0.3070	0.7613
cg04416734	*ALDOA*	0.0675	0.2363	**0.0149**	0.3500
cg14361627	*KLF14*	0.0738	0.2370	**0.0238**	0.3500
cg07583137	*CHMP4C*	0.3030	0.5732	**0.0300**	0.3500
cg18473521	*HOXC4*	0.4300	0.6404	**0.0107**	0.3500
cg20822990	*ATP13A2*	0.4440	0.6475	**0.0355**	0.3518
cg02867102		0.5540	0.7181	**0.0259**	0.3500
cg25428494	*HPSE*	0.7410	0.8280	**0.0402**	0.3518

Age-predictors were calculated for each of the 71 CpGs of the model by Hannum and coworkers and Δ_{age} was used for cox linear regression analysis of overall survival in either the LBC1921 or the LBC1936 study. *P*-values are provided with or without adjustment for multiple testing (71 CpGs) and significant CpGs are highlighted in bold ($P < 0.05$).

Supplementary Table 5: CpGs of the age-predictor by Horvarth that correlate with life expectancy.

**Name**	**Gene Symbol**	**LBC1921 COX *P*-value**	**LBC1921 COX adjusted *P*-value**	**LBC1936 COX *P*-value**	**LBC1936 COX adjusted *P*-value**
cg27319898	ZNF804B	**0.0004**	0.1564	0.3340	0.7651
cg04474832	ABHD14B	**0.0024**	0.4101	0.4220	0.8182
cg06557358	TMEM132E	**0.0092**	0.4374	0.8030	0.9760
cg10266490	ACOT11	**0.0108**	0.4374	0.0682	0.6264
cg07730301	ALDH3B1	**0.0111**	0.4374	**0.0390**	0.5549
cg21801378	BRUNOL6	**0.0116**	0.4374	0.9310	0.9760
cg03270204	DDR1	**0.0135**	0.4374	0.8010	0.9760
cg16150435	C6orf15	**0.0143**	0.4374	0.5260	0.8700
cg06688848	RSPRY1	**0.0145**	0.4374	0.5550	0.8967
cg27494383	LTK	**0.0155**	0.4374	0.8350	0.9760
cg13854874	CHAF1B	**0.0159**	0.4374	0.5230	0.8700
cg02654291	C9orf64	**0.0173**	0.4374	0.2030	0.7608
cg07595943	ADAD2	**0.0174**	0.4374	0.1350	0.7608
cg25411725	SLC22A13	**0.0177**	0.4374	0.3190	0.7608
cg26005082	MIR7-3	**0.0188**	0.4374	0.7630	0.9760

cg24580001	CCDC88B	**0.0210**	0.4581	0.5380	0.8847
cg18139769	SGCE	**0.0284**	0.4930	0.7480	0.9760
cg03588357	GPR68	**0.0293**	0.4930	0.2650	0.7608
cg01511567	SSRP1	**0.0307**	0.4930	0.4890	0.8619
cg19761273	CSNK1D	**0.0323**	0.4930	0.2560	0.7608
cg26614073	SCAP	**0.0342**	0.4930	0.3270	0.7608
cg27544190	C21orf63	**0.0408**	0.4930	0.3690	0.7953
cg17063929	NOX4	**0.0420**	0.4930	0.7920	0.9760
cg01584473	MUC17	**0.0443**	0.4930	0.8850	0.9760
cg06121469	SPG11	**0.0456**	0.4930	0.8390	0.9760
cg25564800	KPNA1	**0.0459**	0.4930	0.9370	0.9760
cg01644850	ZNF551	**0.0467**	0.4930	0.6930	0.9752
cg10281002	TBX5	**0.0473**	0.4930	0.3360	0.7651
cg19273182	PAPOLG	**0.0477**	0.4930	0.7210	0.9760
cg05921699	CD79A	**0.0480**	0.4930	0.2490	0.7608
cg19853760	LGALS1	**0.0482**	0.4930	0.1110	0.7608
cg00168942	GJD4	**0.0486**	0.4930	0.9710	0.9823
cg27016307	HRC	**0.0494**	0.4930	0.6100	0.9337
cg12413566	XIRP1	0.0550	0.4930	**0.0456**	0.5549
cg27377450		0.0725	0.4930	**0.0189**	0.4861
cg19346193	BCCIP	0.0852	0.5040	**0.0013**	0.1640
cg15804973	MAP3K5	0.1120	0.5831	**0.0125**	0.4363
cg07388493	NDUFS5	0.1530	0.6117	**0.0026**	0.1982
cg03891319	ACY1	0.2110	0.6756	**0.0014**	0.1640
cg09646392	TNFSF13B	0.2300	0.7041	**0.0274**	0.5095
cg22920873	C7orf55	0.2360	0.7100	**0.0366**	0.5549
cg14654875	NAT15	0.2590	0.7280	**0.0475**	0.5549
cg01968178	REEP1	0.2880	0.7557	**0.0388**	0.5549
cg10345936	SLC36A2	0.2910	0.7579	**0.0117**	0.4363
cg14329157	WDR69	0.3200	0.7889	**0.0477**	0.5549
cg24126851	DCHS1	0.3380	0.7917	**0.0191**	0.4861
cg19478743	ZMYND15	0.4510	0.8413	**0.0434**	0.5549
cg07770222	C8orf31	0.4650	0.8452	**0.0195**	0.4861
cg10045881	CHI3L2	0.5000	0.8716	**0.0094**	0.4083
cg00864867	PAWR	0.5760	0.8990	**0.0003**	0.1068
cg13038560	C2orf60	0.6250	0.9240	**0.0439**	0.5549
cg22568540	NCRNA00181	0.6460	0.9240	**0.0172**	0.4861
cg03019000	TEX264	0.6870	0.9267	**0.0423**	0.5549
cg21370143	MYBPC3	0.7150	0.9311	**0.0215**	0.4886
cg14658362	RBPMS	0.7180	0.9315	**0.0087**	0.4083
cg07285276	RAPGEF1	0.7820	0.9416	**0.0363**	0.5549
cg19724470	CD274	0.7860	0.9416	**0.0224**	0.4886
cg02479575	MIR7-3	0.8490	0.9497	**0.0028**	0.1982
cg01485645	MLLT6	0.9240	0.9626	**0.0282**	0.5095
cg06952310	NCAN	0.9560	0.9784	**0.0246**	0.5050
cg04268405	CHST3	0.9700	0.9870	**0.0292**	0.5095
cg02489552	CCDC105	0.9940	0.9940	**0.0084**	0.4083

Age-predictors were calculated for each of the 353 CpGs of the model by Horvarth and Δ_{age} was used for cox linear regression analysis of overall survival in either the LBC1921 or the LBC1936 study. *P*-values are provided with or without adjustment for multiple testing and significant CpGs are highlighted in bold ($P < 0.05$). The absence of significant CpGs upon adjustment may partly be due to the higher number of CpGs in this signature (353 CpGs).
